# Supplementary material for: Postural control of arm and fingers through integration of movement commands
Source: eLife. 2020 Feb 11;9:e52507. doi: 10.7554/eLife.52507 (PMC7062460; doi:10.7554/eLife.52507)
Supplement: Supplementary file 1. — Patients completed the Fugl-Meyer Assessment (FMA) and the Action Research Arm Test (ARAT), as well as strength testing in the shoulder and elbow. Strength measurements were repeated twice and the maximal force was recorded on each effort and then averaged across repetitions. Two separate raters scored the FMA and ARAT assessments, and scores were averaged across raters. Missing entries in table indicate that the patient was unable to perform the desired action. Patients were selected based on MRI or CT scans, and/or available radiologic reports. Scans and/or reports were corroborated to determine the level at which the white matter of the corticospinal tract (CST) was lesioned. Here, we provide the level of the brain at which the white matter was lesioned. [file elife-52507-supp1.docx]

**Supplementary Table 1.** Measures of impairment in stroke patients. Patients completed the Fugl-Meyer Assessment (FMA) and the Action Research Arm Test (ARAT), as well as strength testing in the shoulder and elbow. Strength measurements were repeated twice and the maximal force was recorded on each effort and then averaged across repetitions. Two separate raters scored the FMA and ARAT assessments, and scores were averaged across raters. Missing entries in table indicate that the patient was unable to perform the desired action. Patients were selected based on MRI or CT scans, and/or available radiologic reports. Scans and/or reports were corroborated to determine the level at which the white matter of the corticospinal tract (CST) was lesioned. Here we provide the level of the brain at which the white matter was lesioned.

| **ID** | **Age** | **Sex** | **Time since stroke** | **Handed-ness** | **Paretic arm** | **Elbow strength (N)** | | **Shoulder strength (N)** | | **FMA**  **(/66)** | **ARAT**  **(/57)** | **CST lesion location** |
| --- | --- | --- | --- | --- | --- | --- | --- | --- | --- | --- | --- | --- |
|  |  |  |  |  |  | **flexion** | **extension** | **adduction** | **abduction** |  |  |  |
| S001 | 80 | M | 2y | Right | Left | 155.2 (P)  168.1 (NP) | 82.3 (P)  73.0 (NP) | 87.6 (P)  103.2 (NP) | 115.7 (P)  108.5 (NP) | 57.5 | 57 | Right internal capsule |
| S002 | 51 | M | 6y | Right | Left | 177.5 (P)  323.4 (NP) | 114.5 (P)  204.6 (NP) | 229.5 (P)  345.6 (NP) | 152.6 (P)  209.1 (NP) | 40 | 47.5 | Right fronto-parietal white matter |
| S003 | 68 | F | 7y | Right | Right | 142.8 (P)  225.5 (NP) | 99.4 (P)  153.0 (NP) | 128.1 (P)  120.5 (NP) | 107.6 (P)  144.1 (NP) | 34.5 | 19 | Left corona radiata |
| S004 | 30 | F | 5y | Right | Left | 116.1 (P)  157 (NP) | 83.6 (P)  94.3 (NP) | 95.2 (P)  119.7 (NP) | 84.5 (P)  111.2 (NP) | 55.5 | 43.5 | Right precentral and postcentral gyri |
| S005 | 78 | M | 13mo | Right | Right | n/a | n/a | n/a | n/a | 43.5 | 34 | Left corona radiata |
| S007 | 54 | F | 2mo | Left | Right | 140.6 (P)  144.6 (NP) | 101.9 (P)  120.1 (NP) | 107.6 (P)  109.4 (NP) | 97 (P)  85.4 (NP) | 63 | 57 | Left corona radiata |
| S008 | 53 | F | 14mo | Right | Left | 104.5 (P)  136.6 (NP) | 65.4 (P)  148.1 (NP) | 88.1 (P)  133 (NP) | 91.6 (P)  125.9 (NP) | 41 | 25 | Right centrum semiovale |
| S010 | 70 | M | 5y | Right | Left | 90.7 (P)  197.1 (NP) | 68.1 (P)  161.9 (NP) | 177.5 (P)  194.4 (NP) | 82.7 (P)  144.1 (NP) | 20 | 12 | Posterior limb of right internal capsule |
| S011 | 43 | F | 20mo | Right | Right | 116.5 (P)  130.8 (NP) | 97 (P)  109 (NP) | 86.7 (P)  91.6 (NP) | 98.3 (P)  113.9 (NP) | 64 | 57 | Left corona radiata |
| S012 | 48 | M | 6y | Right | Left | 145.9 (P)  358.1 (NP) | 91.2 (P)  207.3 (NP) | 178.8 (P)  292.2 (NP) | 97.4 (P)  226 (NP) | 18.5 | 6.5 | Right corona radiata |
| S013 | 68 | M | 9y | Right | Left | 217.1 (P)  355 (NP) | 112.5 (P)  236.2 (NP) | 220.2 (P)  282 (NP) | 130.3 (P)  174.4 (NP) | 14 | 8 | Right internal capsule |
| S014 | 45 | F | 16mo | Right | Left | 29.8 (P)  114.8 (NP) | 33.4 (P)  113 (NP) | 48.9 (P)  120.5 (NP) | 46.7 (P)  87.2 (NP) | 40 | 39.5 | Right precentral and postcentral gyri |
| S015 | 64 | F | 10y | Right | Left | 149.9 (P)  138.78(NP) | 44 (P)  140.1 (NP) | 73.4 (P)  100.5 (NP) | 48.9 (P)  84.1 (NP) | 22 | 4.5 | Right corona radiata |
| S016 | 38 | F | 21mo | Both | Right | 65.8 (P)  69.4 (NP) | 62.0 (P)  68.4 (NP) | 60.5 (P)  55.4 (NP) | 60.0 (P)  58.6 (NP) | 62.5 | 57 | Left corona radiata |
